# Drug Distribution in Brain and Cerebrospinal Fluids in Relation to IC_50_ Values in Aging and Alzheimer’s Disease, Using the Physiologically Based LeiCNS-PK3.0 Model

**DOI:** 10.1007/s11095-022-03281-3

**Published:** 2022-05-23

**Authors:** Mohammed A. A. Saleh, Julia S. Bloemberg, Jeroen Elassaiss-Schaap, Elizabeth C. M. de Lange

**Affiliations:** 1 Division of Systems Pharmacology and Pharmacy, Leiden Academic Center for Drug Research, Leiden University, Leiden, The Netherlands; 2 PD-value B.V., Houten, The Netherlands

**Keywords:** aging, Alzheimer’s, physiologically based pharmacokinetics

## Abstract

**Background:**

Very little knowledge exists on the impact of Alzheimer’s disease on the CNS target site pharmacokinetics (PK).

**Aim:**

To predict the CNS PK of cognitively healthy young and elderly and of Alzheimer’s patients using the physiologically based LeiCNS-PK3.0 model.

**Methods:**

LeiCNS-PK3.0 was used to predict the PK profiles in brain extracellular (brain_ECF_) and intracellular (brain_ICF_) fluids and cerebrospinal fluid of the subarachnoid space (CSF_SAS_) of donepezil, galantamine, memantine, rivastigmine, and semagacestat in young, elderly, and Alzheimer’s patients. The physiological parameters of LeiCNS-PK3.0 were adapted for aging and Alzheimer’s based on an extensive literature search. The CNS PK profiles at plateau for clinical dose regimens were related to *in vitro* IC_50_ values of acetylcholinesterase, butyrylcholinesterase, N-methyl-D-aspartate, or gamma-secretase.

**Results:**

The PK profiles of all drugs differed between the CNS compartments regarding plateau levels and fluctuation. Brain_ECF_, brain_ICF_ and CSF_SAS_ PK profile relationships were different between the drugs. Aging and Alzheimer’s had little to no impact on CNS PK. Rivastigmine acetylcholinesterase IC_50_ values were not reached. Semagacestat brain PK plateau levels were below the IC_50_ of gamma-secretase for half of the interdose interval, unlike CSF_SAS_ PK profiles that were consistently above IC_50._

**Conclusion:**

This study provides insights into the relations between CNS compartments PK profiles, including target sites. CSF_SAS_ PK appears to be an unreliable predictor of brain PK. Also, despite extensive changes in blood-brain barrier and brain properties in Alzheimer’s, this study shows that the impact of aging and Alzheimer’s pathology on CNS distribution of the five drugs is insignificant.

**Supplementary Information:**

The online version contains supplementary material available at 10.1007/s11095-022-03281-3.

## Introduction

For Alzheimer’s disease (AD) treatment, currently only four small molecule drugs are available that can help reduce the symptoms (1). These include the selective acetylcholinesterase inhibitors donepezil and galantamine, the acetylcholinesterase and butyrylcholinesterase dual inhibitor rivastigmine (for early- to mid-stage AD) (2, 3), and the N-methyl-D-aspartate (NMDA) receptor antagonist memantine (for moderate or severe AD) (4). Cholinesterase inhibitors inhibit the enzyme acetylcholinesterase from breaking down the neurotransmitter acetylcholine into choline and acetate (2, 3). Cholinesterases exist in different forms that can be found in cells, or can be attached to the outer cell membrane (2, 3). Memantine blocks extracellularly the cell membrane bound NMDA receptors (4). Despite their anticipated sites of actions in brain intracellular (brain_ICF_) and/or extracellular (brain_ECF_) fluids, accessible information on AD drug distribution in the human brain is lacking, let aside how this PK profile may be affected by changes in the CNS physiology associated with aging and/or AD. At best, limited data exist on concentrations in subarachnoid cerebrospinal fluid (CSF_SAS_) at the lumbar region, which is often believed to reflect brain_ECF_ concentrations (5–9). Also for AD drug discovery and development, it is important to understand the unbound (brain) target site(s) concentrations, that drive their effects (10). However, assessment of the right information on human brain PK is challenging. First, the best possible direct measurement of unbound drug PK profiles in human brain by microdialysis is limited by ethical restrictions based on the method’s invasiveness. Second, while noninvasive CNS imaging techniques provide crucial information on CNS drug distribution they do not distinguish between the bound and the unbound drug or the parent drug and its metabolites (11). Third, while (invasive) sampling of the lumbar cerebrospinal fluid (CSF) is ethically possible and provides unbound drug concentrations, its use remains limited (5–9), while also it has been shown to be an inaccurate surrogate of brain PK, particularly in the context of CNS diseases (12, 13).

**Fig. 1:**
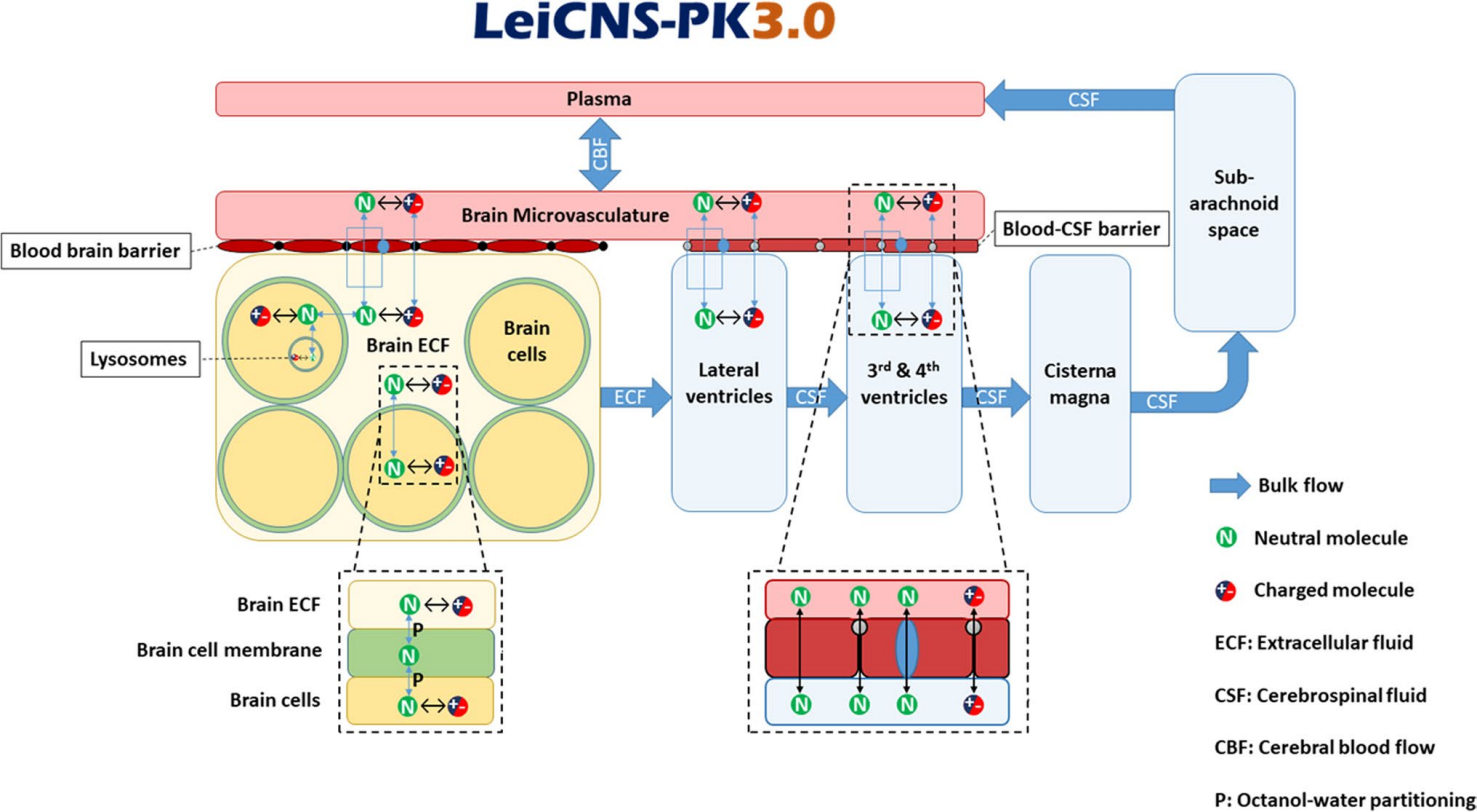
The physiologically based LeiCNS-PK3.0 model structure. This model uses drug physicochemical and biological properties and CNS physiology that together govern the CNS PK of a small molecule drug. This allows the translation of PK predictions in multiple CNS compartments between species and between physiological conditions (health, disease, etc.).

We have previously developed the comprehensive physiologically-based LeiCNS-PK3.0 model (Fig. 1), that has been demonstrated to adequately predict the unbound PK of multiple small molecule drugs in healthy human brain_ECF_ and lumbar CSF_SAS_ (13, 14). The LeiCNS-PK3.0 model accounts for the drug physicochemical properties such as lipophilicity, charge, and molecular weight and for the physiological properties of the human CNS, including the brain_ECF_ and brain_ICF_, and the different CSF compartments, on the basis of the compartments size and surface area. The model accounts for other physiological processes including drug transport across the blood-brain (BBB) and blood-CSF (BCSFB) barrier, physiological fluid flow, intra-extracellular drug distribution, brain tissue non-specific binding, and compartment-specific pH values. The LeiCNS-PK3.0 model can be used to predict the unbound PK profiles at CNS target sites for small molecule CNS drugs and off-target sites for non-CNS drugs and thus predicting potential CNS related toxicities or side effects. In addition, the mechanistic structure of the model allows translation of PK predictions across species but also between the different CNS physiological states, i.e. healthy, diseased, maturing, etc.

Previous studies with the LeiCNS-PK3.0 model have predicted that CNS pathophysiological changes can alter the rate and/or extent of drug transport into the CNS (13–15). These studies addressed the impact of individual CNS pathophysiological changes for multiple small molecule drugs. AD is associated with a complex, multifactorial pathophysiology, which includes but is not limited to brain shrinkage, CSF spatial expansion, brain tissue and cellular composition alteration, and BBB breakdown. Any of these factors has the potential to impact the unbound CNS PK profiles. For a disease (like AD), the impact of disease induced changes on CNS PK should be addressed in combination and not in isolation. Also, AD processes should be distinguished from processes that occur during “normal” aging. Aging represents the best-known risk factor of AD and is associated with similar, but otherwise mild pathophysiological changes (16). Thus, accounting for the pathophysiological changes observed in aging and AD should be performed in a holistic manner to improve the accuracy of CNS PK predictions in these populations (17).

In this paper, we translate the LeiCNS-PK3.0 model to predict the impact of healthy aging and AD-specific pathophysiological changes on brain and CSF PK profiles. The pathophysiological changes associated with each condition were identified from an extensive literature search. The aging and AD versions of the LeiCNS-PK3.0 model will then be used to predict the brain_ECF_ and brain_ICF_ PK profiles of donepezil, galantamine, memantine, and rivastigmine. In addition, two case studies of potential model applications will be performed. In case study 1, the predicted PK profiles of virtual AD patients under chronic treatment with either of the four AD drugs are compared to the relevant unbound IC_50_ at brain_ECF_, brain_ICF_ and CSF_SAS_. CSF_SAS_ includes the lumbar CSF region and, in this sense, represents the most feasible sampling site of the human CNS. In case study 2, the fluctuation of semagacestat PK profiles at brain_ECF_ and brain_ICF_ *versus* CSF_SAS_, and the relation to its IC_50_ is explored. Semagacestat is a gamma secretase inhibitor that failed in clinical trials due to the lack of efficacy and safety concerns (18).

Furthermore, the LeiCNS-PK3.0 model is published as a web-based application at https://cns-pbpk.shinyapps.io/AD-SHINYAPP/ and can used to predict the PK profiles of healthy and AD subjects. In addition, the impact of the pathophysiological changes of brain_ECF_ pH and of paracellular transport on CNS PK can be assessed. These parameters were selected based on the sensitivity analysis results and represent an example of parameters with a drug-dependent impact on CNS PK, while the numerical values are the average change of these parameters in CNS diseases (15).

## Methods

### Translation Strategy

A knowledge-based approach was implemented to translate the predicted PK profiles of cognitively healthy young adult population (CHY) to that of cognitively healthy elderly (CHE) and of AD patients. An extensive literature study of the physiological changes of CNS parameters and processes associated with AD and aging was performed (see Literature Search for details). Results of this literature study were used to inform LeiCNS-PK3.0 parameters.

### Literature Search

An extensive literature search on aging and AD-associated changes in CNS physiology was performed in the PUBMED database (19), with a focus on the parameters that are relevant to parameterization of the LeiCNS-PK3.0 model. Search queries included the terms “Alzheimer’s” or “Aging”, the terms “brain”, “CNS”, etc. and terms related to the CNS physiological parameter in question, for example “cerebrospinal fluid flow”, “blood-brain barrier”. A representation of the search terms used in this literature study is presented in the Supplementary Table 3. In addition, manual forward and backward searches using a seed article were carried out, particularly for CNS parameters with little literature information. Studies including human subjects were selected for further analysis and when humans studies were unavailable, parameter values from animal studies were used. The scaling method of a given parameter, where required, is described in the results section. Where multiple values of the same parameter are found in literature, the mean was calculated weighed by the number of subjects included in the study.

### Aging *Versus* AD

In this study, aging in CHE is defined as the physiological changes that occur in the CNS, from 60 years old onwards, for subjects without cognitive impairment as defined by mini-mental score examination (MMSE) scores. Subjects younger than 60 years old were therefore not considered CHE. Parameter rate of change over age was calculated as the percentage change per year from 60 years old onwards. Where literature information was not suitable for calculating %change per year, the population was divided into 3 categories: young (<60 years old), old (60–75 years old), and older old (>75 years old) and the parameter %change per year was calculated for the parameters of the older categories relative to the young category.

Age as such is not a good marker of AD progression (20), and therefore cognitive scales such as MMSE and clinical dementia rating (CDR) were used to categorize AD patients into mild, moderate, and severe patients (Table I). Information on changes in CNS physiological parameters in moderate-to-severe stages of AD are very rare and therefore we focused on predicting the PK profiles of mild AD patients, which is in line with clinical studies that target the mild AD population. Rate of change of parameters was calculated as the percentage rate of change relative to that in the age matched CHE.

**Table I:** Alzheimer’s Disease (AD) Severity according to CDR, MMSE, and Braak Severity Scores (21–25)

CDR	MMSE	Braak	AD Severity
0	30	0-II	Normal cognition
0.5	26–29	II-III	Questionable
1	21–25	III-IV	Mild
2	11–20	IV-V	Moderate
3	0–10	V-VI	Severe

CDR: Clinical Dementia Rating; MMSE: Mini-Mental State Examination

### LeiCNS-PK3.0

The previously published physiologically based LeiCNS-PK3.0 model (13) was used as the base model that was translated to predict CNS PK profiles in CHE and AD patients. The model structure (Fig. 1) is composed of 9 compartments representing different physiological compartments of the CNS including brain cells and the surrounding extracellular fluid, lysosomes, brain ventricles, cisterna magna, and CSF_SAS_, including lumbar CSF. Plasma PK is used as input into the LeiCNS-PK3.0 model and is typically described by empirical 1-, 2-, or 3-compartment models. Other physiological processes are accounted for in the model such as brain tissue non-specific binding, the actual physiological pH in each compartment to calculate drug ionization as input for ionized and neutral drug transport across cell membranes and across the BBB and BCSFB via paracellular and transcellular routes, and drug transport by bulk fluid flow. Active transport across BBB and BCSFB is accounted for by using the asymmetry factors that are calculated and are translated as described previously (13, 14, 26). Asymmetry factors can be regarded as pure Kp_uu_ values, without influences of other steady state brain processes, for example the constant brain_ECF_ bulk flow. Further details on model equations have been reported previously (13).

The LeiCNS-PK3.0 model input includes drug physicochemical, CNS physiological, and plasma PK parameters, in addition to the unbound tissue-to-plasma partition coefficient across the BBB (Kp_uu,BBB_) and across BCSFB (Kp_uu,LV_ and Kp_uu,lumbar_) (see Table II), which can be obtained from *in vivo* or *in vitro* data. No clinically measured CNS PK data are, thus, required to run the model.

**Table II:** Drug-Specific Parameters

Drug	Donepezil	Galantamine	Memantine	Rivastigmine	Semagacestat
Drug physicochemical parameters (27)					
Molecular mass (g/mol)	379.49	287.35	179.3	250.3	361.4
logP	4.14	1.16	3.31	2.45	0.44
pK_a_	17.02	14.81	NA	NA	11.91
pK_b_	8.62	8.58	10.7	8.89	−3.7
Kp_uu_ and calculated asymmetry factors (AF)^1^					
Kp_uu,BBB_^2^	0.482 (28, 29)	0.826 (30)	2 (31, 32)	0.733 (29)	0.55^3^
AF_in,ECF_	2.1	1	191.3	1	1
AF_ef,ECF_	1	18.4	1	8.6	20.4
Kp_uu,LV_^4^	1.8 (9)	1.2 (33)	0.89 (5)	0.663 (7)	0.55 (34)
AF_in,LV_	1.2	19.5	1	1	1
AF_ef,LV_	1	1	27	10.2	18
Kp_uu,lumbar_^4^	1.8 (9)	1.2 (33)	0.89 (5)	0.663 (7)	0.55 (34)
AF_in,TFV_	1.2	16.4	1	1	1
AF_ef,TFV_	1	1	24.5	10.6	18.6

^1^AF factors are calculated for AD populations

^2^Rat values

^3^Assumed the same as Kp_uu,lumbar_

^4^Human values

#### Physiological Parameters

Physiological parameters represent the CNS physiology in values such as volumes of different compartments, tissue composition, pH of fluids, flows, and transport rates across the membranes (i.e. brain barriers). Physiological parameters of the CHY were as previously described in our work (13). Physiological parameters of CHE and AD patients were calculated using the physiological values of CHY in combination with rates of change as identified from the literature search.

#### Plasma PK Parameters

Parameters of the empirical plasma models of the drugs are available from literature (Table III). Plasma PK parameters that were estimated based on PK data of AD patients were selected when available.

**Table III:** Plasma PK Model Parameters and Dosing Regimens of Different Drugs

Drug	Donepezil	Galantamine	Memantine	Rivastigmine	Semagacestat
Plasma PK model parameters					
Population	Elderly (35)	Alzheimer’s (36)	Alzheimer’s (37)	Alzheimer’s (7)	Volunteers (38)
Number of subjects	129	1089	108	18	14
CL_cen_ (mL min^−1^)^1^	2048	192	228	3333 ^5^	846
Q_cen-per1_ (mL min^−1^)^1^	0	51	0	0	0
V_cen_ (mL)	391,000	157,000	194,000	236,000	71,700
V_per1_ (mL)	0	59,000	0	0	0
K_a_ (min^−1^)	0.022	0.051	0.005	0.052	0.012 (39)
Biological drug properties					
f_u,p_^7^	0.07 (40) ^6,8^	0.83 (40) ^6,9^	0.55 (40) ^6^	0.6 (40) ^6^	0.382 (41) ^2^
f_u,b_^7^	0.107 (42) ^10^	0.333 (42) ^2^	0.071 (43) ^3, 10^	0.376 (42) ^10^	0.413 (42) ^2^
IC_50_ (ng mL^−1^)	0.57 (44) ^4^	55 (44) ^4^	109 (5)	857.2 (44) ^4^	5.4 (18)
Dosing parameters					
Dose (mg)	10	10	20	6	140
Dosing	Once daily	Twice daily	Once daily	Twice daily	Once daily

^1^Apparent values and are corrected for plasma protein binding, i.e. represent unbound drug

^2^Predicted values

^3^Rat values

^4^Corrected for fraction unbound in brain (f_u,b_)

^5^F = 1.4 for 6 mg dose, representing relative bioavailability to 1–5 mg dose

^6^Human values

^7^f_u,p_: fraction of unbound drug in plasma; f_u,b_: fraction of unbound drug in brain

^8^f_u,p_ was determined by ultrafiltration

^9^f_u,p_ was determined by equilibrium dialysis

^10^fu,b was determined by equilibrium dialysis of brain homogenates (45)

#### Kp_uu_ Values

Kp_uu,BBB_, Kp_uu,LV_, and Kp_uu,lumbar_ values are used to calculate the asymmetry factor to account for the active transport of drugs across the BBB and BCSFB. Kp_uu,LV_ and Kp_uu,lumbar_ are calculated based on limited clinical CSF data. Kp_uu,BBB_ is rarely available in humans because of the ethical constraints of the human brain sampling with microdialysis. Therefore, Kp_uu,BBB_ measured with microdialysis in rats, where available, were used to calculate AF_BBB,__rat_ that was translated to AF_BBB_,_human_ based on the decision tree described previously (14). When *in vivo* Kp_uu,BBB_ could not be found, Kp brain measured by brain homogenate was used and converted to Kp_uu,BBB,_ by correcting for plasma protein and brain tissue binding and also for the unequal distribution of charged drug between brain_ECF_ and brain_ICF_ as a result of the pH difference. Equations used to convert Kp to Kp_uu,BBB_ are described in the supplementary materials.

#### Drug Properties

Drug physicochemical properties: molecular weight, lipophilicity (logP), and acid/base ionization constants were available from DrugBank release version 5.1.8 (27) and are presented in Table II. ALOGPS (46) and CHEMAXON (47) were the methods of choice to predict logP and acid/base ionization constants, respectively. Galantamine lipophilicity from the CHEMAXON method was used, as its ALOGPS value was unavailable.

### Sensitivity Analysis

A sensitivity analysis was performed to assess the impact of altered CNS physiology on CNS PK and to support parameter translation where literature information gaps exist. Parameters of the AD model were increased and decreased one-at-time by two and ten folds, except for pH values that were altered by ±1 and ± 2 pH units. The C_max_, T_max_, half-life, and AUC of the altered PK profiles at steady state at the brain_ECF/ICF_ and at the CSF_SAS_ were compared to those of the original profiles.

### LeiCNS-PK3.0 Simulation and Case Studies

The AD and aging versions of LeiCNS-PK3.0 were simulated to assess the impact of aging and AD on steady state PK profiles, i.e. during chronic treatment, at brain_ECF_, brain_ICF_, and CSF_SAS_ as compared to those of CHY. Simulations were performed for drugs that are marketed for AD: donepezil, galantamine, memantine, and rivastigmine. The same plasma PK profile of every drug was used as input for the three populations, in order to isolate the impact of differences in CNS parameters from those of plasma. The AD PK predictions at the brain_ECF_ and brain_ICF_ (the CNS target sites) and the CSF_SAS_ (the CNS sampling site) were, also, compared to the respective unbound IC_50_. *In vitro* IC_50_ values of the four drugs were available from literature. IC_50_ of donepezil, galantamine, and rivastigmine were measured *in vitro* using human brain homogenate (44) and were corrected for brain non-specific binding. IC_50_ of NMDA receptor inhibition by memantine was also quantified *in vitro* using HEK293T cells (48). In addition, a previous analysis performed by de Strooper (18) was revisited to study the fluctuation of semagacestat PK profile at brain_ECF_ and brain_ICF_ *versus* CSF_SAS_ while accounting for the impact of chronic dosing and AD on the PK profiles.

### Software

LeiCNS-PK3.0 simulations were performed in R (version 4.0.3) using the package RxODE (version 0.9.2–0) and the LSODA (Livermore Solver for Ordinary Differential Equations) Fortran package. Literature data were digitized with WebPlotDigitizer version 4.2 (https://apps.automeris.io/wpd/).

## Results

### Literature Findings on CNS Pathophysiology in CHE and AD Patients

An extensive literature search was used to adapt all 35 LeiCNS-PK3.0 parameters to AD- and aging-specific pathophysiology. Results of longitudinal studies on aging-related CNS pathophysiology, where available, were preferable to cross-sectional studies, particularly when studying changes of small magnitude, e.g. brain volume shrinkage (49, 50). Data from cross-sectional designs were extracted from studies with the appropriate control, i.e. CHE *versus* CHY and AD patients *versus* CHE, such that each study would serve as its own control. Mild AD patients represent the major target population of CNS drug development and were therefore the focus of the literature study. Age is a poor marker of AD progression (20), AD severity scores (Table I) were hence used to classify AD patients. Studies comparing AD patients to age-matched CHE were selected to distinguish between aging and AD pathophysiology, unless such studies were unavailable. A summary table of the literature study results is reported in Supplementary Table 4, including relevant references. CNS physiological parameters of CHY, CHE, and AD patients that were used as input to LeiCNS-PK3.0 are reported in Supplementary Table 1.

#### Total Brain Volume

Brain shrinkage begins around 50 years of healthy aging (51, 52). Longitudinal studies reported brain shrinkage as % volume shrinkage /year or as ml volume shrinkage/year, which was converted to % shrinkage/year by normalizing to baseline brain volume. Brain shrinkage rates (in %/year) were not significantly different across the different age groups (results not shown), and hence the mean of brain shrinkage (%/year) across the age groups, weighed by the study size, was calculated as 0.401%/year. The brain of an AD patient shrinks at a faster rate than that of a CHE. Data from cross-sectional studies estimated an average of 5% lower brain volume in AD patients, compared to CHE.

#### Brain_ECF_ and Brain_ICF_ Volume Fraction

Brain_ECF_ and brain_ICF_ volume fractions represent the volume ratio of the brain_ECF_ and brain_ICF_ to total brain, which in healthy conditions are 0.2 and 0.8, respectively (13). Brain_ECF_ volume fraction decreased by 16% in senescent rats (26–32 months) compared to adult rats (2–3 months) and by 26% in senescent mice (17–25 months) compared to 6–8 months mice. Brain_ICF_ volume fraction of the aging, shrinking brain does not change (53). Brain_ECF_ volume fraction increased in mouse AD models compared to age-matched senescent mice by about 40%. No information on brain_ICF_ volume fraction was retrieved and was calculated as the difference of unity and brain_ECF_ volume fraction.

#### Volume of Brain Microvasculature

The volume of brain microvasculature declines significantly with age (54), more in the grey matter than the white matter (55, 56). The ratio of the volume of brain microvasculature to total cerebral blood flow (CBF), however, stays the same with age (54, 57) and the two parameters show a significant, linear correlation (57). In addition, brain microvascular volume to total brain tissue volume stays the same (58). Therefore, brain microvascular volume was calculated to maintain the ratio of brain microvascular volume-to-cerebral blood flow of young age. Similarly, the volume of brain microvasculature does not change in AD patients *versus* CHE and was therefore translated by correcting for the atrophied brain volume.

#### Brain Phospholipid Volume Fraction

Brain tissue non-specific binding of drugs is assumed to occur in LeiCNS-PK3.0 to brain phospholipids. The volume of brain phospholipids is calculated as 5% of the total brain volume and that decreases with age. The decline of the brain phospholipid volume fraction is reported to be biphasic, declining by about 10% in the CHE population between 60 and 80 years old, and further declining by another 8% in the 80–100 years CHE population. The decline rates were calculated as the mean of the values from two studies, weighted by study sample size. The relative volume of different brain structures, e.g. white *versus* grey matter volume, was also accounted for. The fraction of the unbound drug in the AD brain is higher compared to age-matched CHE (43), which is in line with a decrease of the volume fraction of brain phospholipids of 10% on average. The decrease in phospholipids was reported as region-specific (59–61), where it decreases in the cerebellum, frontal cortex and hippocampus, but not in prefrontal cortex and anterior temporal cortex (62, 63). Patients with early onset AD showed a 20% decrease, while late onset AD patients showed no change compared to age-matched CHE (64). The weighted average was calculated considering the differences of the volume of different brain regions, the proportions of the different phospholipids, and the study size.

#### CSF Volume

CSF volume expansion was calculated in a similar fashion as was brain shrinkage. The lateral and 3rd and 4th ventricles were assumed to expand at the same rate, 3.45%/year. The Cisterna magna volume expansion (1.09%/year) was calculated as the extraventricular expansion rate, using the cranial CSF and ventricular expansion rates, considering their relative volumes. The CSF_SAS_ expands at a rate of 0.78%/year. This was calculated as the extraventricular CSF expansion rate as described before and accounting for the contraction of the spinal CSF_SAS_ (65).

Similarly, in AD patients, CSF volume of the ventricles, i.e. lateral, 3rd and 4th ventricles, was assumed to be larger by 39% in AD patients that CHE. Extraventricular CSF, including cisterna magna and cranial CSF_SAS_, expands at a different rate than ventricular CSF and is 21% larger in AD patients compared to CHE. No quantitative information were available on spinal CSF_SAS_ expansion, it can, however, be deduced that it might increase in AD as a consequence of the decrease of spinal cord volume (66), and it was, therefore, assumed to increase at the same rate as cranial CSF_SAS_.

#### Cerebral Blood Flow (CBF)

CBF is reported in literature either as the total CBF (mL/min), representing blood flow to the whole brain, or as normalized CBF, where total CBF is corrected by brain mass (mL/min/100 g brain). Total CBF declines with age (67–71), which is attributed to brain atrophy and not to aging per se (71, 72). Normalized CBF showed no change with age, particularly above 60 years of age (73). Normalized CBF was calculated based on the CHY total CBF and brain volume and was used to calculate the total CBF at different ages, thus correcting for the impact of CHE brain shrinkage on total CBF. In AD patients, normalized CBF decreases compared to CHE in a brain region-dependent manner (74, 75). Normalized CBF is 15% lower in mild AD patients compared to CHE. Total CBF in AD patients was calculated by accounting for the AD- and brain atrophy-related reductions.

#### Brain_ECF_ Bulk Flow

Total brain_ECF_ bulk flow is known to decrease during aging and AD as a result of brain atrophy and other physiological changes including glymphatic system dysfunction, altered aquaporin-4 channel polarization and expression, and amyloid β deposition (76–79). 14C-inulin clearance in mice was reduced in senescent mice (18 months) compared to adult mice (2–3 months) by about 33% (76). Therefore, brain_ECF_ bulk flow, after correction for brain atrophy, was assumed to decrease by about 33% in CHE compared to CHY. Brain_ECF_ bulk flow was shown to decrease by 15% in an AD mouse model compared to wild type mouse and thus brain_ECF_ bulk flow in AD patients was reduced by 15% and was corrected for brain atrophy. Results of the model’s sensitivity analysis suggest that changes in brain_ECF_ bulk flow has no impact on brain_ECF/ICF_ PK profiles.

#### CSF Flow

CSF flow (mL/min) in LeiCNS-PK3.0 model is assumed to have a constant rate across the CSF spaces and is calculated using CSF turnover (day^−1^) and the total CSF volume. CSF production did not differ significantly between CHY and CHE, neither did its flow patterns or velocity at different CSF compartments. There was, however, a small significant increase to CSF outflow with aging. CSF flow is measured at the aqueduct and at the craniocervical junction using MRI. At the aqueduct, CSF flow did not differ significantly with age, except in one study where CHE males showed a 70% higher CSF flow than younger males. At the craniocervical junction, results were contradictory. Two studies showed a decrease of CSF flow with age of about 12.5–25%, while a third study showed about 50% increase in CHE *versus* CHY. CSF production might decrease in AD (80), although this reduction might be an artifact of the measurement technique and not AD per se (81). CSF flow is not altered in AD patients, at both the aqueduct and craniocervical junction. Given the available results, we assumed that CSF flow does not change with increasing age or with AD.

#### Surface Areas of the BBB and BCSFB

Surface area of the BBB represents the surface area of brain microvessels including capillaries and arterioles. BBB SA decreases with aging (82), possibly a result of the observed decrease in capillary density (58, 83, 84), the loss of brain capillaries, and the increase of brain arterioles. The decline of the BBB surface area with aging is reflected by the 10% decrease of the ratio of the brain capillary surface area to brain capillary volume and to brain tissue volume (58). Therefore, total surface area of the BBB was calculated by correcting the CHY BBB surface area for brain atrophy and for the aging-related decrease of 10%.

Direct information on the differences of surface area of the BBB in AD patients compared to CHE was not available. BBB SA can be calculated as the product of the blood vessel’s perimeter, its length, and the capillary number or density. Results of the literature study implied a non-significant change of brain capillary length in AD *versus* CHE (85); a no change to a 5%-increase of capillary diameter; and a no change to 24%-increase of capillary density. Surface area of BBB in AD patients is hence the same or up to 29.3% higher than that in CHE. BBB SA was, hence, corrected for brain atrophy, in addition to an increase of 11.23% compared to CHE.

No information related to the change of BCSFB SA in aging and AD could be found and it was therefore assumed the same in CHY, CHE, and AD patients.

#### Paracellular Transport

BBB paracellular transport represents the drug transport across the torturous paths between the endothelial cells of the BBB. Tight junction proteins between the BBB endothelial cells limit the free passive drug diffusion and reduce the rate of paracellular transport across the BBB. During aging, tight junction protein expression is reduced (86, 87), implying the opening of the BBB and an increase in passive paracellular transport. This effect is counteracted by thickening of the basement membrane, which might reduce passive paracellular transport (86, 87). BBB passive transport is evaluated in the clinic using imaging of gadolinium-based contrast agents. In one study, an increase of BBB passive permeability of about 40% was observed at the hippocampus and caudate nucleus, but not at the superior frontal and inferior temporal gyrus cortex, thalamus, striatum, white matter (WM), corpus collosum, or internal capsule; all these showed no significant difference (88). In another study, an increase of BBB passive permeability of 0.0001%/year or 1.48*10^−12^ min^−1^/year was estimated in grey and white matter (89). Given these data, aging is not expected to impact BBB passive permeability.

Similar to aging, in AD the decrease of tight junction proteins expression and the thickening of the basement membrane impact passive paracellular transport in opposite directions (90). BBB passive paracellular transport, as measured with MRI and contrast agents, demonstrated up to 1.25-, 5-, and 10-fold increase at the hippocampus, grey matter, and cortex, respectively (91). Other regions such as white matter and basal ganglia showed no change of paracellular transport. A mean value of 4.4-fold increase of paracellular transport was used.

Studies comparing the paracellular transport at BCSFB between CHY and CHE and between AD and age-matched CHE were not available in literature. CSF-to-plasma ratio of creatinine and urea showed an increase of 23% and 7%, respectively in AD patients compared to young volunteers (92). Given the small magnitude and the lack of age matching controls in the available study we assumed that paracellular transport at BCSFB is the same in all three populations.

#### BBB Active Transport

The expression and function of Pgp at the BBB in CHE *versus* CHY have been evaluated. Pgp protein and mRNA expression measured with immunohistochemistry showed no significant difference between CHY and CHE populations. Pgp function in CHY *versus* CHE was examined using MR imaging of 11C-verapamil BBB transport and calculating the ratio of the efflux to influx transfer rate constants. Such approach demonstrated that the change of BBB Pgp transport of verapamil ranges from no significant change to about 40% decrease in the Pgp function at the BBB. Interestingly, CHE population demonstrated a higher susceptibility to Pgp inhibition (93). The coadministration of 11C-verapamil and tariquidar resulted in a 30% decrease of Pgp function compared to the administration of solely 11C-verapamil, while Pgp function was not impacted in the young population (93). Collectively, these findings imply that with aging Pgp expression and function do not change, except when a drug is co-administrated with another Pgp substrate or inhibitor. No information could be retrieved on BCRP expression or function at the BBB and its activity was assumed the same in CHE as in CHY.

Information on expression and function of the active transporters, Pgp and BCRP, indicate that BBB active transport might decrease in AD patients. Expression studies of Pgp and BCRP proteins with immunohistochemistry showed a no change to a decrease of expression of 15% and 20%, respectively. Pgp and BCRP protein expression measured with other quantitative techniques such as western blot and LC-MS demonstrated no significant change of the protein expression of both transporters. Studies of BBB Pgp function indicated a no change to a 15–30% decrease of BBB Pgp activity in AD patients. No quantitative information could be retrieved on the changes of active transporters activity and expression at the BCSFB.

The impact of the potential difference of BBB active transporters expression and function on brain PK should be assessed on a drug-by-drug basis, considering the drug’s affinity to a single or multiple active transporter. Donepezil is a substrate of choline transporters (CHT) (94), Pgp, and BCRP (95). No studies could be identified that report on rat-to-human differences in CHT’s expression. Pgp and BCRP protein expression is 0.22- and 1.1-fold different, respectively, in human’s brain microvessels *versus* that of rat (15). The asymmetry factors of donepezil were calculated based on rat Kp_uu,BBB_ and were converted to those of humans by multiplying by 0.22 and 1.1. Galantamine is not a substrate of the major BBB transporters: Pgp, BCRP, MRP4, or of cationic transporters: CHT and OCT; no conversion of asymmetry factors was required. Memantine is a substrate of OCTN1 transporter (96, 97), the expression of which does not change in the brains of AD patients *versus* CHE (98). No information on the rat-to-human differences of OCTN1 expression could be found. Brain-to-plasma drug concentration ratio measured in human was similar to that of rats (99) and therefore asymmetry factors based on rat Kp_uu,BBB_ were calculated. Rivastigmine is a substrate of the CHT (94); the asymmetry factors based on preclinical data were used.

#### Brain_ECF_, Brain_ICF_ and CSF pH

Multiple studies reported a 0.001 unit decrease of brain pH per year of aging (100–103); these studies did not distinguish intracellular and extracellular brain pH. Other studies reported no change of brain extracellular pH (104, 105), which is supported with data from preclinical species, where only brain intracellular pH decreased but not brain extracellular pH (106). Brain intracellular pH was, hence, assumed to decrease by 0.001 pH unit/year, while brain_ECF_ pH stays the same. The pH of CSF of CHE was similar to that of CHY (107).

Studies reported changes in brain pH from pre- and postmortem CHE and AD patients, without discerning intra- or extracellular brain pH. Studies with postmortem data were excluded, as the potential of postmortem brain acidosis increases, particularly with long postmortem-to-tissue collection intervals and in individual with high premortem agony. Changes of brain pH ranged from 0 to an increase of 0.009 pH units, as measured in the brain cortex and hippocampus. The white matter on the other hand decreased by 0.007 pH units in AD patients. No information was available from premortem subjects on cranial CSF pH, which was found to decrease by an average of 0.11 pH units in postmortem samples. Lumbar CSF pH, on the contrary, might increase by 0.018 pH units in AD patients, as compared to healthy young subjects.

### Model Simulations and Case Studies

The LeiCNS-PK3.0 model was used to explore the impact of the pathophysiological changes of aging and AD on the steady state PK profiles of AD drugs at the brain_ECF_, brain_ICF_, and the CSF_SAS_. The parameters of the plasma PK model were based on datasets that included AD patients, except for donepezil, which was based on a CHE population.

#### Aging and AD Have a Minor Impact on Brain_ECF_, Brain_ICF_, and CSF_SAS_ PK Profiles

**Fig. 2:**
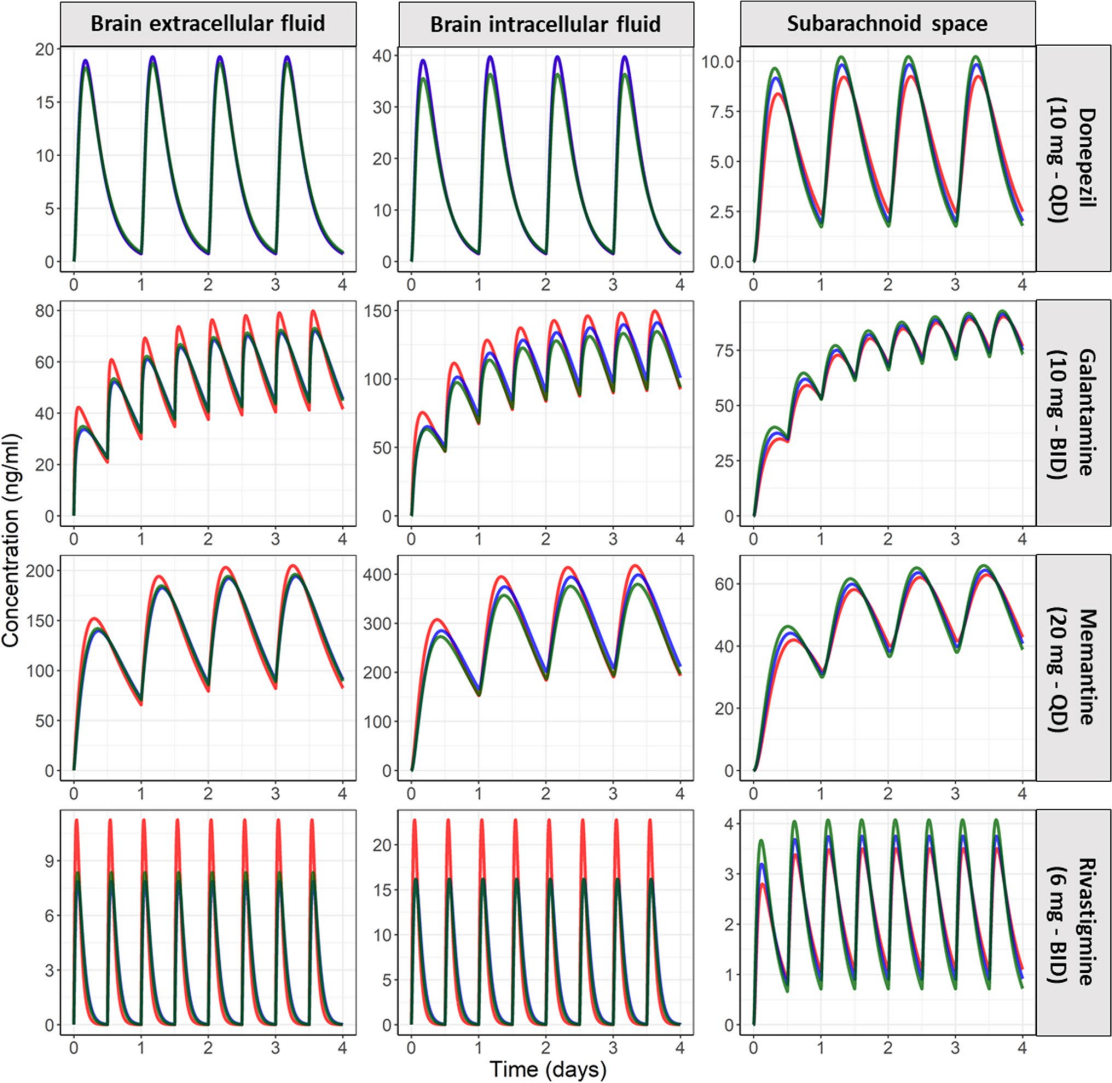
Simulated unbound PK profiles of the four marketed AD drugs at brain_ECF_, brain_ICF_, and subarachnoid space (CSF_SAS_) of CHY (green), CHE (blue), and AD (red) populations. Aging and AD pathophysiological changes have a minor impact on brain_ECF_, brain_ICF_, and CSF_SAS_ PK profiles. Model simulations were performed using the clinical dosing regimens. For each drug, the plasma PK input in the model was based on plasma PK data of CHE or AD patients. Thus, any change of PK profile is attributed to changes of CNS physiology. Please note the different y-axis scale of every panel. Brain_ECF_: brain extracellular fluid, brain_ICF_: brain intracellular fluid, CSF_SAS_: cerebrospinal fluid of the subarachnoid space, CHY: cognitively healthy young adults, CHE: cognitively healthy elderly.

Model simulations of CHY, CHE, and AD populations of the four drugs are depicted in Fig. 2. Brain_ECF_, brain_ICF_ and CSF_SAS_ PK profiles were minimally altered with aging- and AD-related pathophysiological alterations. The change of rivastigmine steady state C_max_, while the most prominent, was less than two-fold.

#### Case Study 1: Brain and CSF_SAS_ PK Profiles Compared to IC_50_ of the Respective Target

**Fig. 3:**
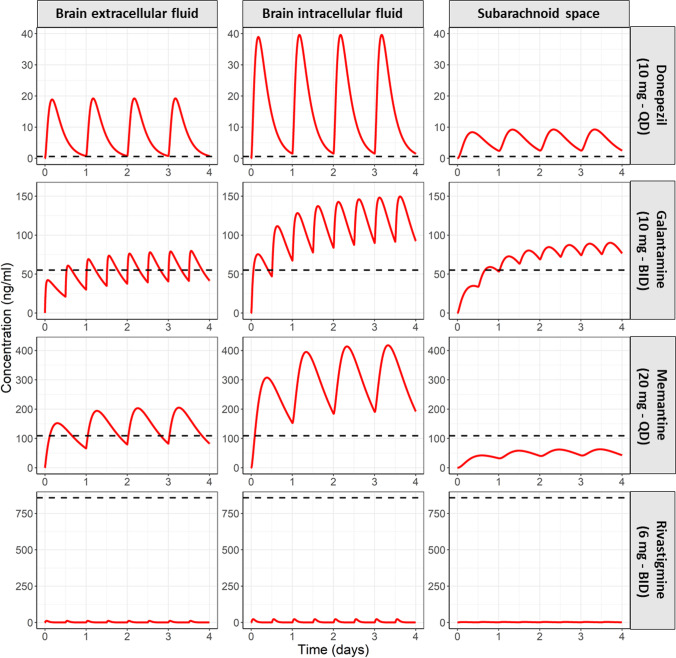
AD predicted PK profiles of the 4 marketed AD drugs at the brain_ECF_, brain_ICF_, and CSF_SAS_ *versus* the IC_50_ of the respective drug target. Target site concentrations are the driver of drug effect and should therefore be evaluated during early stages of drug development. The predicted PK profiles of rivastigmine are below the IC_50_ of acetylcholinesterase. Memantine PK profile at the CSF_SAS_ and not at the brain_ECF_ were lower than the IC_50_ of NMDA receptor, which might imply that lumbar CSF_SAS_ drug concentration is an inaccurate surrogate of that of brain_ECF_.

A comparison between predicted AD PK profile at the brain_ECF_, brain_ICF_, and CSF_SAS_ *versus* the IC_50_ of the respective drug target is depicted in Fig. 3. The brain_ECF_ and brain_ICF_ represent the target site of the cholinesterase inhibitors: donepezil, galantamine, and rivastigmine (108), while brain_ECF_ is the target site of the N-methyl-D-aspartate receptor antagonist, memantine (4). The predicted rivastigmine PK profiles at different CNS locations were consistently below IC_50_, while the brain_ECF_ and brain_ICF_ PK profiles of memantine and galantamine were below the IC_50_ briefly between the doses. The predicted PK profile of memantine at the CSF_SAS_ was below the IC_50_, but not at the brain_ECF/ICF_.

#### Case Study 2: The Importance of Addressing Target Site Concentrations

**Fig. 4:**
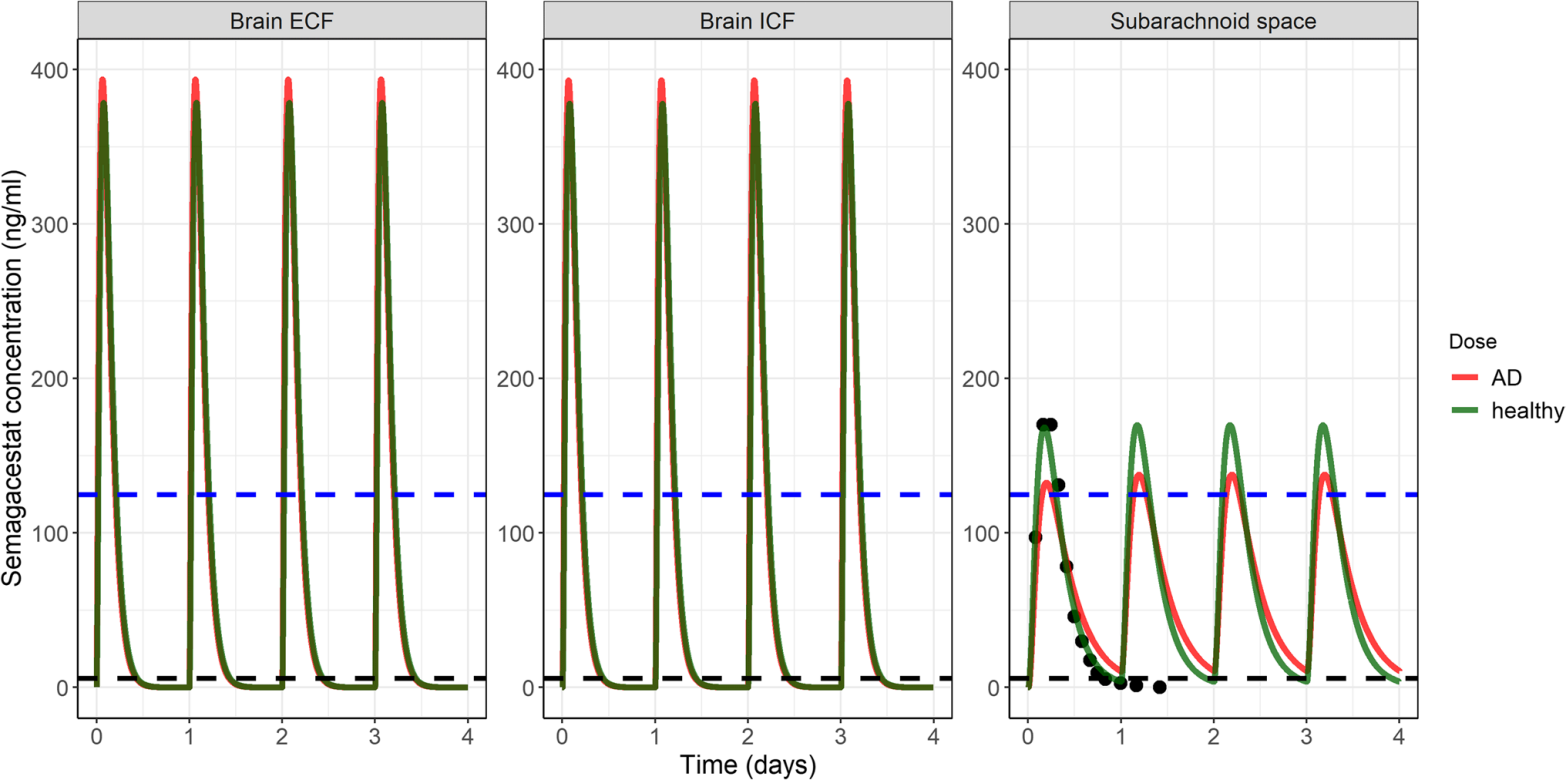
Semgacestat PK profiles of cognitively healthy (CHY) young volunteers (green) and AD patients (red) at the brain_ECF_, brain_ICF_ and at the CSF_SAS_. The black dots in the CSF_SAS_ are semagacestat concentrations at a single dose of 140 mg, measured in CSF samples from CHY volunteers (34). The blue horizontal dashed line represents the paradoxical value used by de Strooper (18) of notch inhibition, while black dashed line represents the IC_50_ of gamma-secretase inhibition by semagacestat. These simulations support the take home messages of the de Strooper (18) analysis on the importance of addressing the fluctuation of the drug concentrations and, in addition, indicate the importance of considering the steady state, potentially disease-altered, PK profiles at the target sites in the brain_ECF_ and brain_ICF_.

The PK profiles of semagacestat in brain_ECF_, brain_ICF_, and CSF_SAS_ of CHY and AD patients are depicted in Fig. 4. Model simulations indicate a higher fluctuation of the PK profile at the brain_ECF_ and brain_ICF_ (C_max_:C_min_ ≈ 9*10^4^) than at the CSF_SAS_ (C_max_:C_min_ ≈ 13). In addition, they show that the brain enters a drug-free period as of 12 hours post dose, unlike CSF_SAS_ PK profiles that are consistently above the IC_50_.

## Discussion

In this study, the CNS PBPK LeiCNS-PK3.0 model was translated to predict the CNS drug distribution of the elderly and AD populations. Model predictions under chronic dosing of the four marketed AD small molecule drugs showed a different pattern of PK profiles fluctuation (C_max_:C_min_) between different compartments. In addition, comparing the predicted PK profiles at the CNS target sites in brain_ECF_ and brain_ICF_ and at the CSF_SAS_ to the IC_50_ value of the respective drug target demonstrated the importance of target site drug concentrations, rather than surrogate compartments, as drivers of drug effect. Interestingly, model simulations showed a little to no impact of AD and healthy aging on the CNS PK profiles, including the target sites.

AD pathophysiology has been studied intensively in humans and in preclinical species, particularly the changes related to BBB integrity but also those related to CBF, brain_ECF_ bulk flow, CSF flow, etc. (109), suggesting the possible alteration of CNS PK. Little, however, is available on the overall impact of the AD pathophysiological changes on CNS PK per se (110). This study is the first, to the best of the authors’ knowledge, to investigate the potential changes of CNS PK associated with healthy aging or AD, showing that both are of little effect. Brain_ECF_ and brain_ICF_ PK profiles of rivastigmine showed the largest difference between CHY/CHE and AD patients, the predicted increase of C_max_ was, however, less than two-fold. We identified the four-fold increase of paracellular transport as the major contribution to the predicted change of rivastigmine brain PK. This was assessed by testing the AD altered parameters values in the model one parameter at a time and observing the parameter’s impact on brain PK (results not shown). These results are in line with a clinical study that demonstrated a minor increase in the exposure of LY2886721 lumbar CSF exposure in AD patients compared to healthy volunteers (111) and with a preclinical study that showed no change of the extent of drug transport across the BBB in a transgenic AD mouse model (112). Taken together, it can be implied that CNS drug concentration measured in young adults might represent that of AD patients. Accounting for the interpopulation differences in physiological characteristics improves brain exposure predictions (113), towards personalized medicine in aging and AD populations (17).

Brain_ECF,_ brain_ICF_, and CSF_SAS_ PK profiles of the four marketed AD drugs were compared to the *in vitro* IC_50_ values of the brain cholinesterases and of the NMDA receptor. The dosing regimens of these drugs were the same as the ones used in the clinic. Brain_ECF_ PK profiles, the target site of the four drugs (4, 108), were above the IC_50_ value, except for rivastigmine. Apart from rivastigmine, these results are expected for successful drugs on the market. Rivastigmine is a dual inhibitor of acetylcholinesterase (IC_50_ = 857.2 ng/ml (44)) and butyrylcholinesterase (IC_50_ = 9.3 ng/ml (114)) and acts at both the brain_ECF_ and brain_ICF_. Brain_ECF_ PK profile at the 6 mg twice daily dosing was below the IC_50_ of both targets. Brain_ICF_ PK profiles was above the IC_50_ of butyrylcholinesterase (Supplementary Fig. 1), the activity of which has been demonstrated to increase with AD progression, in contrast to the activity of acetylcholinesterase, which might decrease (2, 3, 115). Thus, the known therapeutic benefit of rivastigmine can be attributed to dual inhibition of the two cholinesterase enzymes.

The pattern of drug exposure compared to IC_50_ was the same in the CSF_SAS_ and brain_ECF/ICF_ for all drugs, except memantine. Memantine exposure was lower than the IC_50_ at the CSF_SAS_, but not at the brain_ECF/ICF_. This is in line with a previous clinical study, where memantine CSF concentration of the majority of the study subjects was lower than IC_50_, despite an observed clinical effect. This mismatch between the PK profiles at brain_ECF_ and brain_ICF_ and CSF_SAS_ further corroborate previous findings (12, 13) that lumbar CSF is an inaccurate surrogate of brain drug concentrations.

Unestablished target site PK has resulted in as high as one-third of the failures observed in drug development in general (116). Our model predicts the unbound PK of the brain_ECF/ICF_ in CHE and AD patients, by holistically accounting for the associated multifactorial pathophysiology and thus addresses the previously identified PK information gaps and focuses on the AD population that is a prime target population of CNS drug development (90, 110). De strooper (18) identified the learned lessons of a failed clinical trial, studying semagacestat and highlighted the consequences of a fluctuating PK profile on the observed (un)desired drug effect (18). The analysis was, however, performed based on a single dose PK profile from healthy, young volunteers and did not consider the potential impact of AD on CNS PK, the target site PK profile, and steady state PK condition. Our model simulations (Fig. 5) indicate a drastically higher fluctuation of the PK profile at the brain_ECF_ and brain_ICF_ than at the CSF_SAS_, resulting in the different pattern of drug availability of the two compartments. This further highlights the importance of studying target site concentrations as surrogates of drug effect.

Literature information was used to adapt the physiological parameters of LeiCNS-PK3.0 to AD and aging conditions. Comparison of parameter values from different populations across the different studies was avoided where possible, primarily because of different measurement and analysis techniques used in by each study. A clear example was the four orders of magnitude difference of the paracellular permeability calculated as K_trans_ in two different studies (88, 89), which could be attributed to the difference of the imaging protocols, contrast agents, and MR devices. Careful interpretation of heterogeneous literature data on a parameter-by-parameter basis is a crucial requirement to ensure an “as accurate as” possible CNS PK prediction. Meta-analysis studies, performed for each parameter, could provide an unbiased estimate of the parameter mean and the associated variability, further improving the accuracy of model predictions.

A major limitation of this work is that the AD/aging models were not validated against clinical PK data. To the best of the authors’ knowledge, no PK measurements in AD and elderly brain are available. We identified several clinical studies where lumbar CSF PK profiles were measured in AD patients on chronic treatment with either donepezil, memantine, or rivastigmine (5–9). The data were, however, inadequate for model validation either because of the missing sampling time after the donepezil dose (9), the unrealistically higher plasma and CSF donepezil and memantine concentrations at the end of the dosing interval (5, 6), or the unavailability of population plasma PK profile of rivastigmine (7, 8). Another limitation related to the knowledge-based translation approach is that the accuracy of the PK predictions is reliant on the extent and quality of available literature. Literature studies on few parameters were either missing, inaccurate, or contradictory and might reduce the reliability and accuracy of the model. For example, no literature reports could be identified on AD- or aging-related changes of lysosomal volume, lysosomal de-acidification, surface area and the paracellular transport of the blood-CSF barrier. To address this drawback, a sensitivity analysis of the AD model was performed (Supplementary Fig. 2) and indicated that these parameters do not have a major impact on the major target site, i.e. brain_ECF_ PK profile and were therefore assumed the same as the healthy condition (13). In addition, contradictory results were found regarding changes of CSF flow in AD, ranging from no change to an increase in AD patients compared to CHE. CSF flow does not impact the brain_ECF_ PK profiles, but does impact the sampling site, i.e. lumbar CSF, and might result in inaccurate implication regarding the rate of drug removal from the CNS. Addressing the knowledge gaps and inaccuracies of AD-related pathophysiology would further improve the model’s reliability. The model as currently presented, thus, cannot yet replace preclinical and clinical studies. LeiCNS-PK3.0 nevertheless is suited to support early stages of drug development, mainly in initial drug screening and design and analysis of first-in-human trials.

The LeiCNSPK3.0 model provides insights of small molecule drug PK of brain_ECF_ and brain_ICF_ in AD patients, and can therefore help in optimizing and accelerating the development of small molecule drugs for AD. To date, the marketed small molecule drugs have been approved for merely the symptomatic management of AD. Emerging multitarget treatment approach have shown potential as disease modifying agent and potential treatment of AD. This can be either by polypharmacy (i.e. combining multiple drugs) (117) or by multi-target-directed ligand (i.e. single drug acting on multiple targets) (118). To this end, in silico methods are useful to explore the therapeutic advantages of this multitarget approach. For example, combining our model (i.e. PK component) with a quantitative systems pharmacology model (i.e. pharmacodynamic component) of AD disease pathways will allow the exploration of possible interaction of drug target site exposure (in case of polypharmacy) or effect (117).

## Conclusion

In this study, a literature-based approach was used to translate the CNS PBPK LeiCNS-PK3.0 model to predict the CNS PK profile of elderly and AD populations. Steady state brain_ECF_ PK predictions of donepezil, galantamine, and memantine were above the respective IC_50_. Fluctuations of the PK profile of semagacestat showed distinct patterns in brain compared to CSF_SAS_. CNS PK profiles were comparable among CHY, CHE, and AD patients implying a minor impact of healthy aging and AD on CNS PK, including the target sites.

LeiCNS-PK3.0 is available as a web-based application (https://cns-pbpk.shinyapps.io/AD-SHINYAPP/) that can be used to predict CNS PK profiles of CHY and AD populations, in addition to the impact of selected pathophysiological changes on CNS PK.

## Supplementary Information

ESM 1 (DOCX 738 kb)

ESM 2 (XLSX 71 kb)
