# Supplementary material for: Drug Distribution in Brain and Cerebrospinal Fluids in Relation to IC50 Values in Aging and Alzheimer’s Disease, Using the Physiologically Based LeiCNS-PK3.0 Model
Source: Pharm Res. 2022 May 23;39(7):1303–19. doi: 10.1007/s11095-022-03281-3 (PMC9246802; doi:10.1007/s11095-022-03281-3)
Supplement: Supplementary file 1 — (DOCX 738 kb) [file 11095_2022_3281_MOESM1_ESM.docx]

**Supplementary materials to “Drug distribution in brain and cerebrospinal fluids in relation to IC_50_ values in aging and Alzheimer’s disease, using the physiologically based LeiCNS-PK3.0 model****”**

Mohammed A. A. Saleh^1^, Julia S. Bloemberg^2^, Jeroen Elassaiss-Schaap^2,3^, Elizabeth C. M. de Lange^4^

1. Division of Systems Pharmacology and Pharmacy, Leiden Academic Center for Drug Research, Leiden University, Leiden, The Netherlands. ORCID ID: 0000-0002-0517-6051

2. Division of Systems Pharmacology and Pharmacy, Leiden Academic Center for Drug Research, Leiden University, Leiden, The Netherlands.

3. PD-value B.V., Houten, The Netherlands. ORCID ID: 0000-0002-3333-861X

4. Division of Systems Pharmacology and Pharmacy, Leiden Academic Center for Drug Research, Leiden University, Leiden, The Netherlands. ORCID ID: 0000-0001-8303-1117

Correspondence to email: [ecmdelange@lacdr.leidenuniv.nl](mailto:ecmdelange@lacdr.leidenuniv.nl), telephone: +31 71 527 6330

Running title: predicting brain PK in AD and aging using translational modeling

**Keywords:** Alzheimer’s, aging, physiologically based pharmacokinetics

**Supplementary figures**


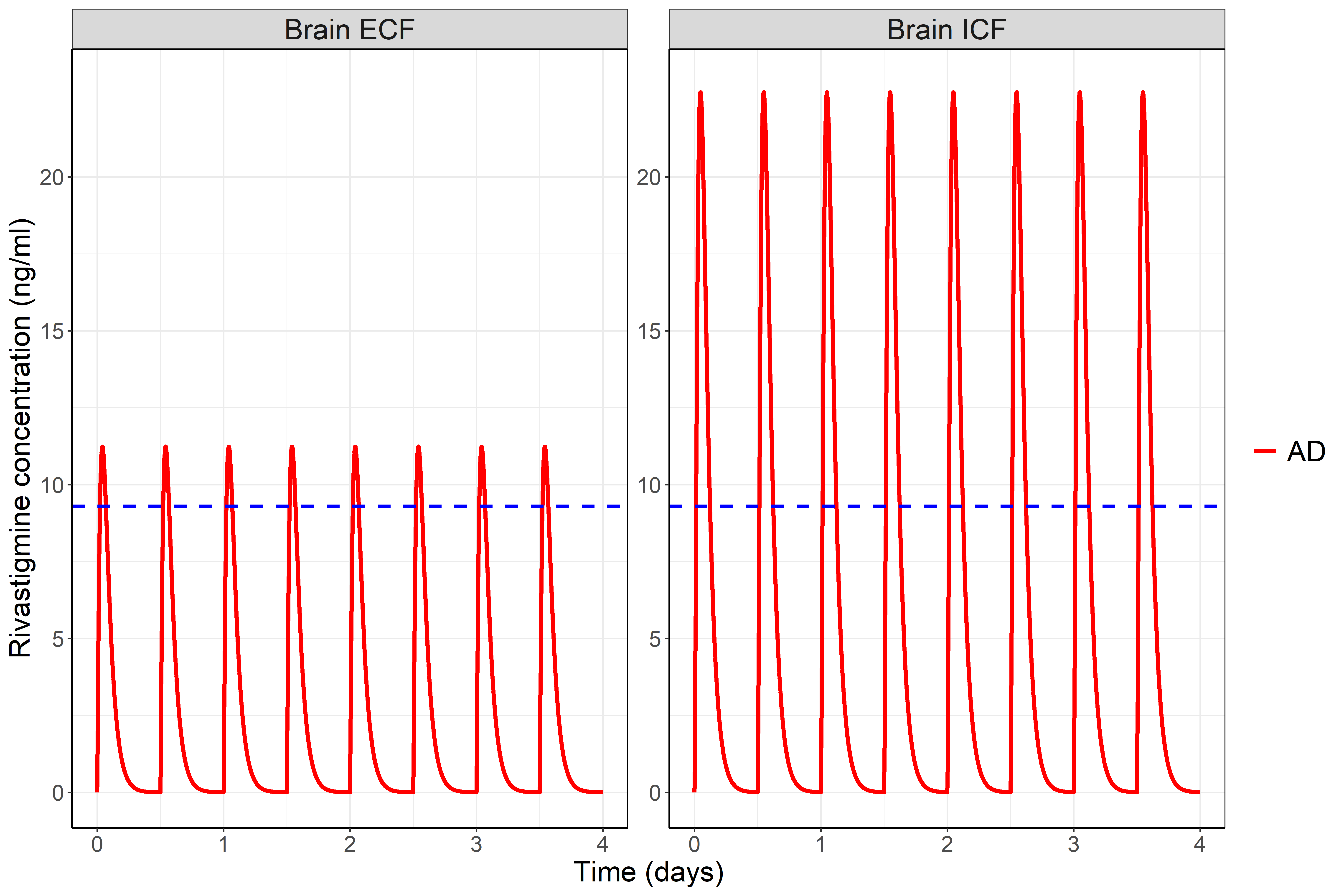


Supplementary figure 1: AD predicted PK profiles of rivastigmine (6 mg, twice daily) at brain_ECF_ and brain_ICF_ versus the IC_50_ of butyrylcholinesterase. The blue dashed line represents the IC_50_ value of butyrylcholinesterase. The predicted PK profiles of rivastigmine are below the IC_50_ of acetylcholinesterase but exceed that of butyrylcholinesterase at the brain_ECF/ICF_.

**Sensitivity analysis of the AD version of LeiCNS-PK3.0**

A sensitivity analysis was performed on the AD version of LeiCNS-PK3.0 for donepezil, galantamine, memantine, and rivastigmine. Model parameters were altered by two and ten folds, while pH values were changed by one and two pH units. The pharmacokinetic parameters: C_max_, T_max_, AUC, and half-life were used to assess the impact of parameters alterations on PK profiles at the compartments of interest: brain_ECF_, brain_ICF_, and CSF_SAS_. Sensitivity analysis results are depicted in supplementary figure 2. The PK profiles of the compartments of interest were not impacted by changes of ventricular volume, brain microvasculature volume, brain_ECF_ bulk flow, CSF pH, and the surface area of blood-CSF barrier. Changes of related to CSF parameters: CSF flow and volumes of the SAS and of the cisterna magna affected the CSF but not brain_ECF_ and brain_ICF_ PK profiles, which is in line with our previous results (1). The other parameters affected the PK profiles depending on the drug’s physicochemical properties. PK changes due pH depended on the acidic and basic ionization constants of the drug. Those due to cerebral blood flow and volume fraction of phospholipids relied on the drug’s lipophilicity, evident by the notable change observed for lipophilic drug, donepezil (logP = 4.14). PK parameters of the more hydrophilic drugs, rivastigmine and galantamine, were impacted by changes of the brain cell and lysosomal surface area. Changes of surface area of the BBB and that of BBB paracellular transport affect the PK profiles depending on the paracellular-to-transcellular drug transport ratio, which is determined according to the drug’s molecular weight and lipophilicity and if the drug is actively transported at the BBB.

Supplementary figure 2: sensitivity analysis of the AD LeiCNS-PK3.0. Parameters (top) were varied (bottom) by two and ten folds, while pH values were changed by one and two pH units. The final profiles at the brain_ECF/ICF_ and CSF_SAS_ (right) were evaluated according to the changes in the PK parameters (left): C_max_, T_max_, AUC, and half-life. The magnitude of change in percentage of pharmacokinetic parameters is given by the color scale (right), where blue, red, and white represent increase, decrease, and no change, respectively. pHCSF: pH of cerebrospinal fluid, pHECF: pH of brain extracellular fluid, pHICF: pH of brain cells, pHLYS: pH of brain lysosomes, pHMV: pH of brain microvasculature, PPA-BBB: effective surface area of paracellular transport at the blood-brain barrier, PPA-CSF: effective surface area of paracellular transport at the blood-CSF barrier, QCBF: cerebral blood flow, QCSF: cerebrospinal fluid flow, QECF: brain_ECF_ bulk flow, SABBB: blood brain barrier surface area, SABC: surface area of brain cell membrane, SACSFB: surface area of blood-CSF barrier, SALYSO: lysosomal surface area, VCM: volume of cisterna magna, VECF: volume of brain extracellular fluid, VICF: volume of brain cells, VLV: volume of lateral ventricles, VLYS: volume of lysosomes, VMV: volume of brain microvasculature, Vphb: volume fraction of brain phospholipid, VSAS: subarachnoid space volume, VTFV: volume of third and fourth ventricles.


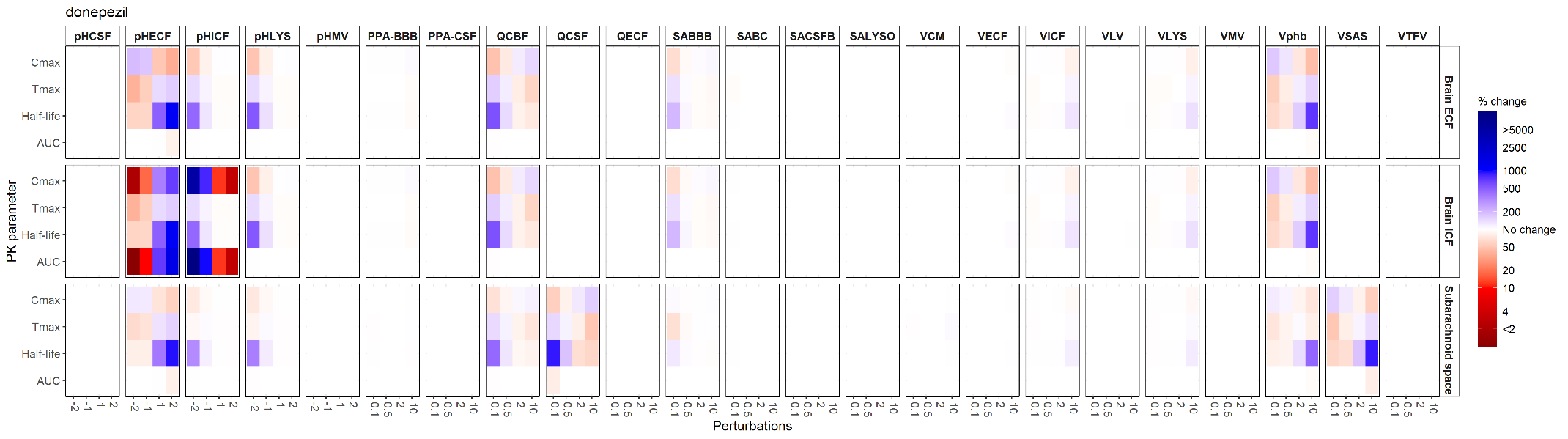


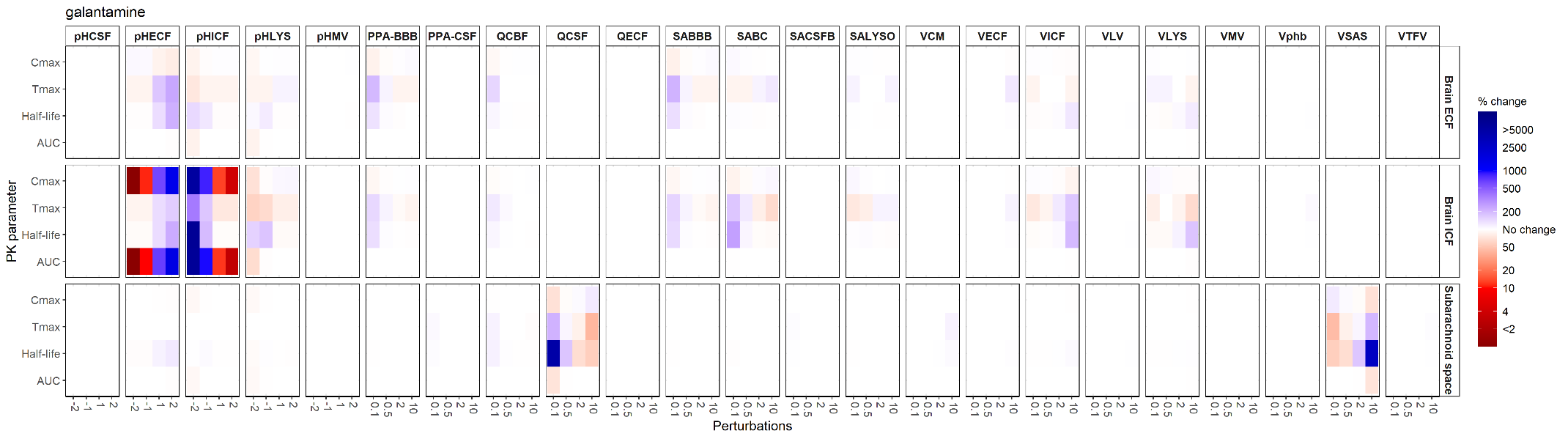


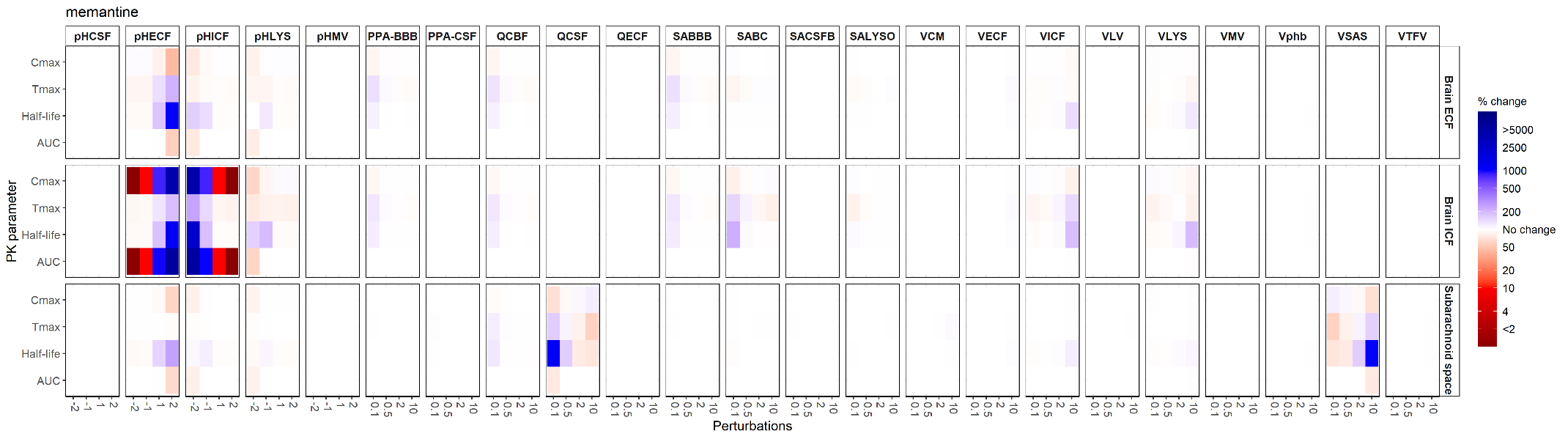


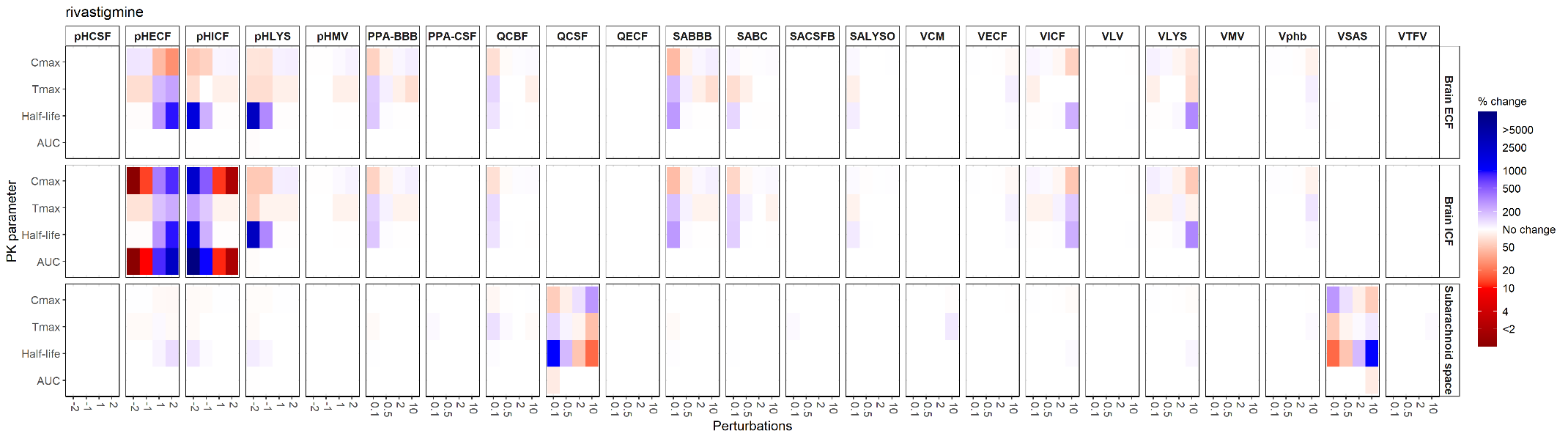


**Supplementary equations to convert Kp_brain_ into Kp_uu,BBB_**

These equations are used to convert Kp_brain_ to Kp_uu,BBB,_ by correcting for plasma protein and brain tissue binding and also for the unequal distribution of charged drug between brain_ECF_ and brain_ICF_ as a result of the pH difference. The following assumptions were made. Active transport is not present at brain cells level or at lysosomes. Unbound drug exists in the brain extracellular and intracellular fluids and in lysosomes, and drug can bind to the phospholipids of the brain cell membrane.

**Definitions**

C_brain_: brain concentration as measured by homogenate methods

A: amounts

ECF: brain ECF

ICF: brain ICF

LYS: lysosomes

BCM: brain cell membrane

V_br_: brain volume

Cp: total plasma concentration

C_P,u_: unbound plasma concentration

f_u,p_: unbound fraction of plasma

V_u,Br_: unbound volume of distribution in brain as measured by brain slice method

WT_Br_: brain weight

PHF: neutral drug fraction

**Equations**

In the presence of experimentally measured Vu,br,

Kp_uu,BBB_ = Kp*(1/fu,p)*(V_br_ / (WT_Br_*V_u,Br_))

If experimentally measured V_u,Br_ is not available,

$$C_{brain}= \frac{A_{ECF}+A_{ICF}+ A_{LYS}+A_{BCM}}{V_{br}}$$

$$C_{brain}= \frac{C_{ECF}*V_{ECF}+C_{ICF}*V_{ICF}+ C_{LYS}*V_{LYS}+C_{BCM}*V_{BCM}}{V_{br}}$$

At Steady State,

C_ECF_*PHF_ECF_ = C_ICF_*PHF_ICF_ = C_LYS_*PHF_LYS_

$$P_{oct/water}= \frac{C_{BCM}}{C_{ECF}*{PHF}_{ECF}}$$

Every C_x_ in terms of C_ECF_,

$$C_{brain}= \frac{C_{ECF}*V_{ECF}+C_{ECF}*\frac{{PHF}_{ECF}}{{PHF}_{ICF}}*V_{ICF}+ C_{ECF}*\frac{{PHF}_{ECF}}{{PHF}_{LYS}}*V_{LYS}+C_{ECF}*{PHF}_{ECF}*P_{Octanol/Water}*V_{BCM}}{V_{br}}$$

Every V_X_ in terms of V_Br_

V_ECF_ = 0.2*V_Br_; V_ICF_ = 0.74*V_Br_; V_LYS_ = 0.01*V_Br_; V_BCM_ = 0.05*V_Br_

$$C_{brain}= \frac{C_{ECF}*{0.2*V}_{Br}+C_{ECF}*\frac{{PHF}_{ECF}}{{PHF}_{ICF}}*0.74*V_{Br}+ C_{ECF}*\frac{{PHF}_{ECF}}{{PHF}_{LYS}}*0.01*V_{Br}+C_{ECF}*{PHF}_{ECF}*P_{\frac{O}{W}}*0.05*V_{Br}}{V_{br}}$$

Taking C_ECF_ as common factor, V_Br_ cancels each other out,

$$C_{brain}=C_{ECF}*(0.2+\frac{{PHF}_{ECF}}{{PHF}_{ICF}}*0.74+ \frac{{PHF}_{ECF}}{{PHF}_{LYS}}*0.01+{PHF}_{ECF}*P_{\frac{O}{W}}*0.05)$$

Dividing both sides by C_P_,

$$\frac{C_{brain}}{C_{P}}=\frac{C_{ECF}}{C_{P}}*(0.2+\frac{{PHF}_{ECF}}{{PHF}_{ICF}}*0.74+ \frac{{PHF}_{ECF}}{{PHF}_{LYS}}*0.01+{PHF}_{ECF}*P_{\frac{O}{W}}*0.05)$$

$Cp=\frac{C_{p,u}}{f_{u,p}}$ || $Kp=\frac{C_{brain}}{C_{P}}$

$$Kp=\frac{f_{u,p}*C_{ECF}}{C_{P,u}}*(0.2+\frac{{PHF}_{ECF}}{{PHF}_{ICF}}*0.74+ \frac{{PHF}_{ECF}}{{PHF}_{LYS}}*0.01+{PHF}_{ECF}*P_{\frac{O}{W}}*0.05)$$

$$Kp,uu,BBB=\frac{C_{ECF}}{C_{p,u}}$$

$Kp={Kp}_{uu,BBB}*f_{u,p}*(0.2+\frac{{PHF}_{ECF}}{{PHF}_{ICF}}*0.74+ \frac{{PHF}_{ECF}}{{PHF}_{LYS}}*0.01+{PHF}_{ECF}*P_{\frac{O}{W}}*0.05)$

**Supplementary table 1: CNS physiological parameters of cognitively healthy young, cognitively healthy elderly, and Alzheimer’s disease patients**

| **Parameter** | | **Adults** | **75-elder** | | **AD** | |
| --- | --- | --- | --- | --- | --- | --- |
|  |  | **value** (1) | **value** | **%^1^** | **value** | **%^1^** |
| **Volume (mL)** | **Total brain** | 1251 | 1131 | 90.4 | 1081 | 86.5 |
|  | **Brain extracellular fluid (brain_ECF_)** | 254 | 181 | 71.3 | 247 | 97.3 |
|  | **Brain intracellular fluid (brain_ICF_)** | 1001 | 905 | 90.4 | 834 | 83.4 |
|  | **Brain cell lysosomes (VLYS)** | 13 | 11 | 90.4 | 10 | 83.4 |
|  | **Lateral ventricles** | 20 | 47 | 233.3 | 65 | 324.3 |
|  | **3rd and 4th ventricles** | 3.0 | 7.0 | 233.3 | 9.8 | 324.3 |
|  | **Cisterna magna** | 1.0 | 1.3 | 131.2 | 1.6 | 158.8 |
|  | **Subarachnoid space** | 116 | 141 | 121.4 | 170 | 146.9 |
|  | **Brain microvasculature** | 46 | 41 | 90.4 | 34 | 74.1 |
| **Flow (mL/min)** | **Brain bulk flow** | 0.20 | 0.15 | 72.4 | 0.20 | 98.7 |
|  | **CSF flow** | 0.42 | 0.42 | 100 | 0.42 | 100 |
|  | **Cerebral blood flow (CBF)** | 689 | 623 | 90.4 | 510 | 74.1 |
| **Surface area (cm^2^)** | **Blood–brain barrier (SABBB)** | 150000 | 121962 | 81.3 | 129695 | 86.5 |
|  | **Blood CSF barrier (SABCSFB)** | 15000 | 15000 | 100 | 15000 | 100 |
|  | **Brain cell membrane (SABCM)** | 2666517 | 2511324 | 94.2 | 2379051 | 89.2 |
|  | **Lysosomes membrane** | 1980260 | 1809922 | 91.4 | 1668827 | 84.3 |
| **width (µm)** | **Blood brain barrier Blood** | 0.5 | 0.5 | 100 | 0.5 | 100 |
|  | **Blood CSF barrier** | 0.5 | 0.5 | 100 | 0.5 | 100 |
| **pH** | **Plasma and brain MV** | 7.4 | 7.4 | 100 | 7.4 | 100 |
|  | **Brain extracellular fluid (pHECF)** | 7.3 | 7.3 | 100 | 7.309 | 100.1 |
|  | **Brain cells (pHICF)** | 7 | 6.975 | 99.6 | 6.984 | 99.8 |
|  | **Brain cell lysosomes** | 5 | 5 | 100 | 5 | 100 |
|  | **Cerebrospinal fluid** | 7.3 | 7.3 | 100 | 7.19 | 98.5 |
| **Effective**  **surface area (%)** | **BBB Transcellular transport** | 0.998 | 0.998 | 100 | 0.998 | 100 |
|  | **BCSFB Transcellular transport** | 0.998 | 0.998 | 100 | 0.998 | 100 |
|  | **BBB paracellular transport** | 0.00004 | 0.00004 | 100 | 0.00018 | 444.8 |
|  | **BCSFB paracellular transport** | 0.00016 | 0.00016 | 100 | 0.00016 | 100 |
| **Volume  fraction** | **Brain phospholipids** | 0.0565 | 0.0513 | 90.8 | 0.0469 | 82.9 |
|  | **Brain_ECF_** | 0.2 | 0.16 | 80.0 | 0.2284 | 114.2 |
|  | **Brain_ICF_** | 0.8 | 0.8 | 100 | 0.7716 | 96.4 |
|  | **Lysosomes** | 0.0125 | 0.0125 | 100 | 0.0125 | 100 |
| **Count** | **Total brain cells (Nbr.cells)** | 1,71E+11 | 1,71E+11 | 100 | 1,71E+11 | 100 |

^1^Compared to adults

**Supplementary table 2. Age versus aging stage of different species**

| **Species** | **Stage** | **Age** | **Age units** |  |
| --- | --- | --- | --- | --- |
| Mouse | young | 3-6 | month | (2) |
| Mouse | middle aged | 10-15 | month |  |
| Mouse | old | 18-26 | month |  |
| rat | young | 6-12 | month | (3,4) |
| rat | middle aged | 18-24 | month |  |
| rat | old | 30-36 | month |  |
| human | young | 20-30 | year | (2) |
| human | middle aged | 38-47 | year |  |
| human | old | 59-69 | year |  |

**Supplementary table 3: Examples of the different search queries used in the literature study**

| **CNS parameter** | **Search queries** |
| --- | --- |
| **Aging** | |
| Brain volume | "brain" AND ("volume" OR "structure" OR "shrinkage") AND ("elderly"OR "aging" OR "age" "old"); |
| Brain microvascular volume | ("cerebral" OR "brain") AND ("blood volume" OR "vascular volume" OR "microvasculature" OR "microvascular") AND ("volume") AND ("aging") |
| Cerebral blood flow | ("cerebral blood flow" [tiab]) AND ("aging" [tiab] OR "ageing"[ti] OR "age"[ti]) |
| Cerebrospinal fluid flow | ("CSF flow"[tiab] OR "cerebrospinal fluid flow"[tiab] OR "CSF flows"[tiab] OR "cerebrospinal fluid flows"[tiab]) AND ("aging"[tiab] OR "elderly"[tiab] OR "age"[title] OR "ageing"[tiab]) |
| CSF pH | ("Aging" OR "AGE" OR "ELDERLY" OR "AGEING") AND ("CSF" OR "cerebrospinal fluid") AND "pH" |
| Ventricular volume | ("ventricles volume" OR "ventricular volume"[tiab] OR "cerebrospinal fluid volume"[tiab] OR "CSF volume"[tiab]) AND ("aging"[tiab] OR "ageing"[tiab] OR "elderly"[tiab]) |
| BBB Pgp | ("central nervous system" OR "CNS" OR "brain" OR "blood-brain barrier" OR "BBB" OR "blood brain barrier") AND ("aging" OR "ageing" OR "Elderly") AND ("p-gp" OR "p-glycoprotein" OR "pgp" OR "permeability glycoprotein") |
| BBB BCRP | ("CNS" OR "central nervous system" OR "brain" OR "blood-brain barrier" OR "BBB" OR "blood brain barrier") AND ("aging" OR "ageing" OR "Elderly") AND ("BCRP" OR "breast cancer Resistance protein" OR "ABCG2") |
| BBB MRP4 | ("CNS" OR "central nervous system" OR "brain" OR "blood-brain barrier" OR "BBB" OR "blood brain barrier") AND ("aging" OR "ageing" OR "Elderly") AND ("multidrug resistance protein" OR "ABCC4" OR "MRP4") |
| BBB OAT/OCT | ("CNS"[tiab] OR "central nervous system"[tiab] OR "brain"[tiab] OR "blood-brain barrier"[tiab] OR "BBB"[tiab] OR "blood brain barrier"[tiab]) AND ("aging"[tiab] OR "ageing"[tiab] OR "Elderly"[tiab]) AND ("OAT"[tiab] OR "OCT"[tiab] OR "organic anionic transporter"[tiab] OR "organic cationic transporter"[tiab]) NOT ("retina" OR "retinal") |
| Blood-CSF barrier active transport | ("CP" OR "choroid plexus" OR "cerebrospinal fluid" OR "CSF" OR "blood-cerebrospinal" OR "BCSFB") AND ("aging" OR "ageing" OR "Elderly") AND ("p-gp" OR "p-glycoprotein" OR "pgp" OR "permeability glycoprotein" OR "BCRP" OR "breast cancer Resistance protein" OR "ABCG2" OR "multidrug resistance protein" OR "ABCC4" OR "MRP4") |
| BBB paracellular transport | ("paracellular") AND ("BBB" OR "blood-brain barrier" OR "blood brain barrier") AND ("aging" OR "ageing" OR "elderly" OR "senescence") |
|  | ("blood-brain barrier"[tiab] OR "blood brain barrier"[tiab] OR "BBB"[tiab]) AND ("permeability"[tiab]) AND ("aging"[tiab] OR "ageing"[tiab] OR "elderly"[tiab]) |
| Non-specific binding | phospholipids[tiab] AND "brain"[tiab] AND "aging"[tiab] |
| Brain_ECF_ fraction | (brain) AND ("interstitial" OR "extracellular") AND ("aging" OR "ageing") AND ("fraction") |
| Brain_ICF_ fraction | (brain OR CNS OR "central nervous system") AND ("aging" OR "ageing" OR "elderly" OR "elder" OR "senescence" OR "senescent") AND ( "volume fraction" OR "volume ratio") AND ("intracellular" OR "cellular") |
| BBB surface area | ("brain"[tiab] OR "cerebral"[tiab]) AND ("aging"[tiab] OR "ageing"[tiab] OR "elderly"[tiab] OR "elder"[tiab] OR "senescence"[tiab] OR "senescent"[tiab]) AND ("vascular volume" OR "microvascular volume" OR "vascular area" OR "microvascular area" OR "vascular density" OR "microvascular density") |
| Blood-CSF barrier surface area | ("choroid plexus"[tiab] OR "blood cerebrospinal fluid barrier"[tiab] OR "cerebrospinal fluid barrier"[tiab] OR "blood CSF barrier"[tiab] OR "BCSFB"[tiab]) AND ("aging"[tiab] OR "ageing"[tiab] OR "age"[tiab] OR "senescence"[tiab] OR "elderly"[tiab]) AND ("surface area" OR morphology[tiab]) |
| **Alzheimer’s disease** | |
| Brain microvascular volume | (alzheimer's[tiab] OR alzheimer[tiab]) AND (brain[tiab] OR cerebral[tiab]) AND ("vascular volume"[tiab] OR "vasculature volume"[tiab] OR "microvascular volume"[tiab] OR "blood volume"[tiab]) |
| Brain_ICF_ fraction | (intracellular OR cell) AND ("volume ratio" OR "volume fraction") AND (alzheimer's OR alzheimer) |
| Blood-CSF barrier surface area | ("choroid plexus"[tiab] OR "blood cerebrospinal fluid barrier"[tiab] OR "cerebrospinal fluid barrier"[tiab] OR "blood CSF barrier"[tiab] OR "BCSFB"[tiab] OR "blood-cerebrospinal fluid barrier"[tiab] OR "blood-CSF barrier"[tiab]) AND ("alzheimer"[tiab] OR "alzheimer's"[tiab]) AND ("surface area" OR morphology[tiab] OR structure[tiab] OR length[tiab] OR villi[tiab] OR pathophysiology[tiab]) |
| Blood-CSF barrier paracellular transport | ("choroid plexus"[tiab] OR "cerebrospinal fluid barrier"[tiab] OR "blood CSF barrier"[tiab] OR "BCSFB"[tiab] OR "blood-cerebrospinal fluid barrier"[tiab] OR "blood-CSF barrier"[tiab]) AND ("alzheimer"[tiab] OR "alzheimer's"[tiab]) AND (permeability OR paracellular OR gadolinium) |
| Brain_ECF_ fraction | ("extracellular"[tiab] OR "interstitial"[tiab]) AND ("brain"[tiab]) AND ("volume"[tiab]) AND ("alzheimer"[tiab] OR "alzheimer's"[tiab]) |
| BBB surface area | ("alzheimer's"[tiab] OR "alzheimer"[tiab]) AND ("brain microvessels"[tiab] OR "brain microvascular"[tiab] OR "cerebrovascular"[tiab] OR "blood-brain barrier"[tiab] OR "blood brain barrier"[tiab]) AND ("surface area"[tiab] OR "density"[tiab] OR "diameter"[tiab]) |
| Cerebral blood flow | ("cerebral blood flow"[tiab] OR "brain blood flow"[tiab]) AND ("Alzheimer's" [tiab] OR "Alzheimer" [tiab] OR "AD" [tiab]) |
| Paracellular transport | ("paracellular") AND ("BBB" OR "blood-brain barrier" OR "blood brain barrier" OR "Blood CSF Barrier" OR "BCSFB") AND ("Alzheimer's" OR "Alzheimer" OR "AD") |
| CSF volume | ("ventricles volume"[tiab] OR "ventricular volume"[tiab] OR "cerebrospinal fluid volume"[tiab] OR "CSF volume"[tiab]) AND ("alzheimer's"[tiab] OR "alzheimer"[tiab]) NOT ("heart" OR "cardiac") AND ("MRI" OR "magnetic resonance ") |

**Supplementary references**

1. Saleh MAA, Loo CF, Elassaiss-Schaap J, De Lange ECM. Lumbar cerebrospinal fluid-to-brain extracellular fluid surrogacy is context-specific: insights from LeiCNS-PK3.0 simulations. J Pharmacokinet Pharmacodyn. 2021;48(5):725–41.

2. Flurkey K, Currer JM, Harrison DE. Mouse Models in Aging Research. Mouse Biomed Res. 2007;3:637–72.

3. Quinn R. Comparing rat’s to human’s age: How old is my rat in people years? Nutrition. 2005;21(6):775–7.

4. Andreollo NA, Freitas E, Araújo MR, Lopes LR. Review Article Rat’ s Age Versus Human’ S Age : What Is The Relationship? Arq Bras Cir Dig. 2012;25(1):49–51.
